# 

**Published:** 1869-03-01

**Authors:** 


### CHICAGO MEDICAL JOURNAL

J. ADAMS ALLEN, M.D., LL.D., Editor. WALTER HAY, A.M., M.D., Associate Editor.

All letters for THE JOURNAL should be addressed to DR. J. ADAMS ALLEN, No. 71 Dearborn Street, Chicago, Ill.

TERMS—$3 PER ANNUM, IN ADVANCE.

$4 if not paid before 1st of July, in each year. Single Copies, 25 Cents.

### *Annual Announcement of the Spring Term for 1869.*

The Session will commence WEDNESDAY, March 3d, 1869, and continue to the 1st of July.

The Faculty will be assisted by the following gentlemen:

W. R. MARSH, M. D., Instructor in the Principles and Practice of Medicine.

J. H. ETHERIDGE, M. D., Instructor in Materia Medica.

C. T. PARKES, M.D., Instructor in Anatomy.

HENRY M. LYMAN, M. D., Instructor in Physiology.

CURTIS T. FENN, M. D., Instructor in Obstetrics.

J. N. DANFORTH, M. D., Instructor in Toxicology and Medical Jurisprudence.

H. F. CHESBROUGH, M. D., Demonstrator of Anatomy.

W. C. HUNT, M. D., Instructor in Microscopical Anatomy and use of Microscope.

---

The Course will consist of daily lectures and recitations at the College, and Clinical observations in the Hospitals, Infirmaries, and Dispensaries of the City.

PROF. BLANEY will give instruction in Practical Chemistry, affording access for that purpose to the large and well-appointed Laboratory of the College.

PROF. POWELL will teach Surgery.

PROF. GUNN will conduct his Surgical Clinique at the College.

PROF. ROSS will conduct the Medical Clinique and instruct in Physical Diagnosis and Diseases of the Chest.

The County Hospital will afford two Medical and Surgical Cliniques each week.

The Marine Hospital will be open to students of the Course.

The Charitable Eye and Ear Infirmary is located near the College and will be open every day of the week for Clinical teaching, by PROF. HOLMES, Lecturer on Diseases of the Eye and Ear.

The College Dispensary will be open every day, affording to students an excellent opportunity for perfecting themselves in the art of diagnosis.

The Dissecting Room will be kept open during the entire session, and will be abundantly supplied with material for the study of Practical Anatomy.

Special instruction in Practical Chemistry will be given by Prof. Blaney, to such as desire.

Graduates of the College will be admitted to the Spring Session on the matriculation ticket. All others will be required to procure tickets.

Fees for the Spring Term—exclusive of Hospital and Demonstrator's Tickets —$20.

Certificates of attendance upon the Full Course will be considered the equivalent of time passed under the instruction of a medical practitioner during the Spring and Summer months of the year.

Every student of medicine is urged to consider the advantages to him of remaining in the city during the spring and summer, when the best opportunities are afforded for successful study. The hospitals and dispensaries are then overrunning with material illustrative of disease, and the student may have these facilities at a comparatively trivial expense.

He needs access to libraries and reading rooms; and, above all, he needs to become familiar with earnest men in the profession, that his character may be stamped by the example of their industry and habits of attention. He needs a text-book, daily and hourly illustrated in the street, the class-room, the laboratory, the hospital ward, and the dead-house, and the active rivalry of associates—a sure road to excellence.

The design of the Course is that it may supply the want of a numerous body of students who must pass the time more or less unprofitably while removed from the best appliances during a long vacation. The majority of medical students in the Northwest do not need to be reminded of the value of time and money. A year given to close observation under the tutelage of the College will insure practical ability with prospect of immediate reward. The College offers to become Preceptor as well as *Alma Mater*, that the highest lustre may be given its *Almuni*.

---

For the Annual Catalogue of the College, and for additional information concerning the Spring Session,

Address,

Dr. E. POWELL, *Treas., 45 S. Clark St.*

Students arriving in the City are requested to call at once upon the Janitor, Mr. CHARLES KEIL, at the College Building, corner of North Dearborn and Indiana streets, Chicago.

---

The Twenty-seventh Annual Session of the regular Course of Lectures will commence on Wednesday, September 29th, 1869, and continue eighteen weeks.

